# Nutrient Analysis of Raw and Cooked USDA Prime Beef Cuts

**DOI:** 10.3390/nu16172912

**Published:** 2024-08-31

**Authors:** Emma G. Mortensen, Hannah F. Fuerniss, Jerrad F. Legako, Leslie D. Thompson, Dale R. Woerner

**Affiliations:** 1 Department of Animal and Food Sciences, Texas Tech University, Lubbock, TX 79406, USA; jerrad.legako@ttu.edu (J.F.L.); leslie.thompson@ttu.edu (L.D.T.); dale.woerner@ttu.edu (D.R.W.); 2 Texas Beef Council, Austin, TX 78726, USA; hannah@txbeef.org

**Keywords:** beef, nutrients, fatty acids, prime beef, nutrition, beef quality

## Abstract

Nutrient composition data that accurately represent available beef products are critical to understanding beef’s role in healthy dietary patterns. The quality of beef products has changed over the past several decades, and updated nutrient data are warranted as USDA Prime beef cuts become more available. In an effort to provide a complete nutrient profile for frequently purchased USDA Prime beef cuts, five USDA Prime cuts; strip loin steak, tenderloin steak, ribeye steak, top sirloin steak, and rib roast were collected from retail stores in six geographical locations over three collections for macro- and micronutrient analysis in both the raw and cooked state. The separable lean portion of all analyzed USDA Prime cuts qualified as a good or excellent source, providing 10–19% or at least 20% of the daily value, respectively, for protein, niacin, vitamin B12, selenium, phosphorus, and zinc per FDA labeling claim standards. There was not a significant difference in cholesterol content between any of the cuts, raw or cooked (*p* ≥ 0.44 and 0.34, respectively). The percent lipid in raw, separable lean portions of the rib roast and strip loin steak was significantly greater than the lipid portion in tenderloin and top sirloin steaks (*p* ≤ 0.01). Per USDA standards, the separable lean portions of tenderloin steak and top sirloin steak qualify as lean beef, containing less than 10 g total fat, less than 4.5 g saturated fat, and less than or equal to 95 mg cholesterol. The current study provides the most up-to-date nutrient analysis for USDA Prime beef cuts, helping consumers and health professionals better identify the role of high-quality beef cuts in healthy dietary patterns.

## 1. Introduction

The beef industry continues to respond to consumer demand for consistently high-quality beef [1]. To produce the highest quality product, beef production and processing must align with consumer preferences for tenderness, flavor, and juiciness, as well as nutritional expectations. If tenderness is acceptable, flavor is weighted as the most important palatability trait [2,3,4]. Flavor tends to improve with a greater percentage of intramuscular fat, or marbling [5,6,7]; conversely, most nutritional expectations favor a leaner product. Because these demands have created antagonistic expectations, data are needed to evaluate the nutrient profile of highly marbled beef products.

Beef is recognized as a nutrient-dense, complete protein [8] that inherently provides numerous essential vitamins and minerals; however, since the first Dietary Guidelines for Americans (DGA) in 1980, each report, released every five years, has continually recommended a decrease or moderation in red and processed meat consumption. Early recommendations were established as a result of the saturated fat and cholesterol content of red meat. Saturated fat has in the past been associated, primarily in observational research, with increased low-density lipoprotein levels and potential risks for cardiovascular health; however, emerging evidence has also found no beneficial effects of limiting saturated fats, particularly when consumed in the context of a whole food matrix [9,10]. Because saturated fats are a topic of ongoing debate and investigation amongst nutrition scientists, epidemiologists, health professionals, and public health agencies, more research is needed to isolate red meat consumption from other potentially confounding lifestyle and dietary factors that could affect morbidity and mortality.

Food Data Central, previously known as the National Nutrient Database for Standard Reference, provides nutrient information for developing nutrition labels, supplying nutrient data for nutrition monitoring surveys and other nutrition research, guiding the decisions of health professionals, and shaping the educational content of dietitians and nutritionists [11]; therefore, it is important that USDA Prime beef be accurately analyzed and available. Nutrient analysis of beef products is necessary to characterize the composition of beef products for consumers, retailers, and health professionals. Furthermore, public and commercially available nutrient databases used for analysis of intakes in research studies also benefit from updated compositional analysis.

The composition of beef cuts can change as the quality of beef improves. The percentage of cattle in the United States graded as USDA Prime has increased from 3.3% in 2000 to 9.3% in 2023 [12]. The USDA defines Prime beef as beef produced from young, well-fed beef cattle with an abundant amount of marbling, or interspersed fat within the lean meat [13]. Increased marbling is sought after for its benefits to flavor, but the additional fat impacts the fatty acid content and composition of the beef cut. Nutrient composition data of USDA Choice beef, which represents about three-quarters of the industry, are well studied and represented in nutrient databases. The increasing prevalence of USDA Prime beef cuts in the retail and food-service markets emphasizes the need for accurate nutrient composition data to allow health professionals, retailers, and consumers to understand the nutritional profile of prime beef cuts and to make informed decisions.

The objective of this study was to build a complete nutrient profile of frequently purchased beef cuts from USDA Prime beef carcasses, on a raw and cooked basis, to provide updated and accurate information to retailers, consumers, educators, and health professionals. Additionally, nutrient values were compared across cut types to highlight the variety of high-quality available beef cuts.

## 2. Materials and Methods

### 2.1. Experimental Design

Strip loin steaks, tenderloin steaks, top sirloin steaks, ribeye steaks, and rib roasts (North American Meat Processors (NAMP) Meat Buyer’s Guide #1180, 1190A, 1184B, 1112A, and 112A, respectively) [14] labeled as USDA Prime were collected from retail grocers, wholesale clubs, or local meat markets in six United States cities (Chicago, Illinois; Denver, Colorado; Lubbock, Texas; Miami, Florida; San Luis Obispo, California; and Washington, District of Columbia). Product collections were designed to represent regional differences in conventional, retail beef products. Times of collection were scheduled across ten months to account for seasonal differences in beef production. At each of the collection locations, four steaks and two roasts were obtained for each of the specified cuts. Two steaks were designated for raw analysis, and two steaks were designated for cooked analysis. Similarly, one roast was used for raw, and one roast for cooked analysis. Beef was purchased directly from the case or retail counter to represent common consumer purchasing methods. A total of 180 consumer-available beef products were obtained over three collection periods, from six geographical locations (N = 180, 5 cuts × 6 cities × 3 collections × 2 cooked vs. raw).

Product was vacuum sealed, if not already, and shipped overnight to the Texas Tech University Gordon W. Davis Meat Laboratory in a Styrofoam cooler and box with frozen gel ice packs. Upon arrival at Texas Tech University, boxes were received and inspected for proper packaging and integrity of samples. Product was frozen at −20 °C until dissection and analysis.

### 2.2. Cooking of Retail Cuts

Strip loin steaks, tenderloin steaks, top sirloin steaks, ribeye steaks, and rib roasts from each collection period and location were designated for cooked analysis. Prior to cooking, all cuts were thawed at 0 to 4 °C for 24 to 48 h; thaw time was dependent on the thickness of steaks and roasts. Once internal temperature reached 0 to 4 °C, each cut was removed from the bag, blotted dry, and weighed to the nearest 0.01 g on an Adam Equipment benchtop scale (Adam Equipment Inc., Oxford, CT, USA). Internal temperature was recorded using a thermocouple thermometer and probe (Cooper Atkins Corporation, Gainesville, FL, USA). Rib roasts were oven-roasted while the strip loin steaks, tenderloin steaks, top sirloin steaks, and ribeye steaks were pan-grilled. Standard roasting protocols were adapted from Acheson et al. (2015) [15] for all rib roasts. Each roast was placed on a non-stick, metal rack in a Calphalon roasting pan (Calphalon LLC., Perrysburg, OH, USA). A consumer, gas-powered Thor range oven, Model #HRG3080ULP (Thor Kitchen Inc., Ontario, CA, USA) was preheated to 160 °C. A thermocouple thermometer probe (Cooper Atkins Corporation, Gainesville, FL, USA) was inserted in the geometric center of each roast and two roasts were placed on the middle rack of the oven. Roasts were cooked until an internal temperature of 65 °C, or medium–well cooking designation by USDA standards, was achieved. Roasts were removed, and three temperatures were recorded: initial pull temperature, peak temperature, and final temperature. Final temperature and weight were recorded at 30 min post cooking. Roasts were covered and refrigerated at 0 to 4 °C for at least 12 h, before cooked dissections. For pan grilling, a porcelain-enameled cast iron skillet, model # 80131/058DS (Tramontina USA Inc., Sugar Land, TX, USA) was preheated until the surface temperature reached 195 °C, as read by an infrared thermometer (Milwaukee Tool, Brookfield, WI, USA). Two steaks were cooked together to represent one experimental unit. Before grilling, steaks were weighed to the nearest 0.01 g for initial cut weight, and internal temperature was recorded using the thermocouple thermometer and probe (Cooper Atkins Corporation, Gainesville, FL, USA). Commercially available non-stick canola and coconut oil cooking spray was very lightly sprayed in the bottom of the pan to prevent sticking. Two steaks were placed in the geometric center of the skillet and cooked for 7 min on each side, then flipped every subsequent 2 min until an internal temperature of 71 °C, or well-done designation per USDA standards, was achieved. A thermocouple thermometer (Cooper Atkins Corporation, Gainesville, FL, USA) was used to determine internal temperature. Steaks were removed from the pan, weighed, and the final temperature was recorded before being placed on a wire rack. Cooked steaks were covered and refrigerated at 0 to 4 °C for at least 12 h before cooked dissections.

### 2.3. Cut Dissections

For raw cut dissections, beef cuts were thawed at 0 to 4 °C for 24 to 48 h, depending on cut thickness. Dissections were conducted by previously trained Texas Tech University meat science personnel in the absence of direct light to protect the light-sensitive vitamins from degradation in a 0 to 4 °C environment using powder-free nitrile gloves. A weight was taken of each cut and package to the nearest 0.01 g, the package was removed, and the cut was weighed again to the nearest 0.01 g for initial cut weight. Plastic-coated freezer paper sheets were weighed and placed on a cutting tray. Cuts were dissected using disposable, stainless steel scalpels (Integra Miltex, York, PA, USA) into separable lean, seam fat, external fat, and refuse components. Each dissected component, as well as the freezer paper, was weighed, and a weigh back of components was calculated. A final weigh-back tolerance of 98 to 100% was established before dissections, and any cut that did not meet the pre-established weigh-back tolerance was removed from the study and replaced with another product of the same cut from the same location. Cooked cut dissections were completed using the same protocol as listed above. The only difference was that the cooked cuts were held at 0 to 4 °C for 12 h prior to dissection.

Dissection components were defined as follows: separable lean included all lean, edible muscle tissue, intramuscular fat (marbling), and edible connective tissue; seam fat included adipose tissue oriented between lean tissue; external fat consisted of adipose tissue around the external edges of lean tissue; and refuse was comprised of heavy, inedible connective tissue, and bone. For cuts lacking seam fat, external fat, and refuse, dissection was not required, so separable lean weight was used for the total sample weight.

### 2.4. Homogenization

The protocol for homogenization was adapted from Acheson et al. (2015) [15]. Homogenization occurred in the absence of direct light with the use of powder-free nitrile gloves. Immediately following dissection, separable lean components of each cut were cubed into approximately 2.5 cm^3^ pieces and submerged into liquid nitrogen using a stainless-steel bowl and strainer until completely frozen, then transferred to a 7-quart (6.62 L) Robot Coupe BLIXER 6V (Robot Coupe USA Inc., Ridgeland, MS, USA), and blended until the sample was completely homogenized to a fine powder. Each sample was blended for about 10 s on low speed (1500 rpm) and 30 s on high speed (3500 rpm) until finely powdered. Homogenized samples were placed in Whirl-Pak bags (Whirl-Pak, Madison, WI, USA) using a stainless-steel spoon cooled in liquid nitrogen. Seam and external fat samples were also homogenized and transferred to Whirl-Pak bags (Whirl-Pak, Madison, WI, USA). Dissected, separable lean components from each individual cut (strip loin steaks, tenderloin steaks, top sirloin steaks, ribeye steaks, and rib roasts) were homogenized individually. Seam and external fat from all cuts were combined for each collection period and homogenized together, keeping raw and cooked fat separate. Refuse was discarded and not included in homogenization. Homogenized sample bags were stored at −80 °C until further compositing and analysis.

### 2.5. Compositing Scheme

**Figure 1:**
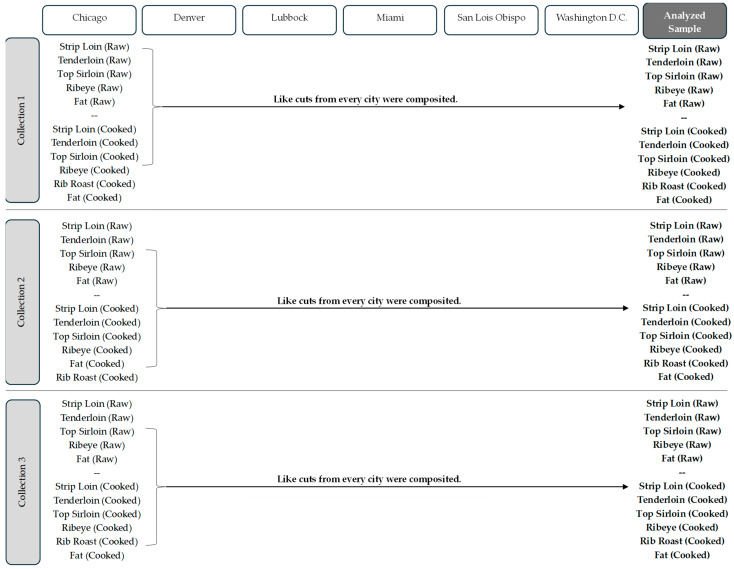
Compositing scheme for separable lean portions of raw and cooked beef cuts, as well as the dissected fat samples (combined external and seam fat from all cuts).

The compositing protocol for raw and cooked samples was the same but conducted at different times to prevent cross-contamination of samples. Ribeye steak and rib roast were composited together for all raw, separable lean samples but were composited separately for cooked samples because of different cooking methods (pan grilling versus roasting, respectively). Following homogenization, the separable lean portion of like cuts, from each of the six locations, was composited together per collection period. Because there were three collection periods, there were a total of three composited samples per cut (n = 3 for each cut and cook type). Seam and external fat from all cuts from all collection locations were homogenized together to create one composite fat sample for each collection period (n = 3 raw fat samples and n = 3 cooked fat samples). There were a total of 12 composited raw samples (4 cuts × 3 collections), 15 composited cooked samples (5 cuts × 3 collections), and six composited fat samples (raw and cooked for each of 3 collections). The compositing scheme is depicted in Figure 1.

Compositing was conducted by combining equal weights in a 7-quart (6.62 L) Robot Coupe BLIXER 6V (Robot Coupe USA Inc., Ridgeland, MS, USA) and transferring to Whirl-Pak bags. All samples were vacuum sealed in Whirl-Pak bags and stored at −80 °C until further analysis.

### 2.6. Nutrient Analysis

Nutrient analyses were conducted at USDA Agricultural Research Service (ARS) approved facilities including the Animal and Food Sciences Laboratory at Texas Tech University in Lubbock, Texas, and commercial external laboratories. Proximate values (total protein, fat, moisture, and ash), fatty acid content (37 fatty acids), cholesterol content, mineral content (calcium, magnesium, potassium, sodium, iron, zinc, copper, manganese, selenium, and phosphorus), B vitamin content (B6, B12, niacin, riboflavin, thiamin, pantothenic acid), as well as vitamin A, D (D_2_ and D_3_), E, and K (K_1_) content were measured for each cut and fat sample on a raw and cooked basis.

National Institute of Standards and Technology (NIST) standard reference material 1546a Meat Homogenate (Gaithersburg, MD, USA) was used to validate precision, and blind duplicates with calculated coefficient of variation were used to determine accuracy of nutrient values determined at Texas Tech University and external laboratories.

#### 2.6.1. Protein Analysis

Crude protein was determined using the AOAC official method 992.15 (2006) [16], which utilized a nitrogen determinator (TruSpec CN Carbon/Nitrogen Determination; Laboratory Equipment Corporation (LECO), St. Joseph, MI, USA) at Texas Tech University. Approximately 0.3 g ± 0.01 g of homogenized sample was weighed into ceramic boats and was combusted on the LECO mass spectrometer to determine nitrogen concentration. Percent protein was approximated by multiplying the percent nitrogen by a factor of 6.25.

#### 2.6.2. Moisture Analysis

Moisture analysis was performed using the AOAC oven drying method 950.46 (1995) [17] at Texas Tech University. Approximately 5.0 ± 0.02 g was weighed out into acid-washed, dried, and pre-weighed crucibles and allowed to dry for 24 h at 100 °C in a forced air-drying oven. Percent moisture (%MC) was calculated using the formula listed below:

%MC = [(wet weight − dry weight) ÷ wet weight] × 100

#### 2.6.3. Ash Analysis

Ash was determined using the ashing method described in the AOAC official method 923.03 (1995) [18] at Texas Tech University. Approximately 5.0 ± 0.02 g of sample was placed into an acid-washed, dried, pre-weighed crucible. Samples were dried for 24 h at 100 °C, weighed, then placed into a Thermolyne box (Thermo Fisher Scientific, Waltham, MA, USA) furnace at 550 °C for 18 h. Percent ash was calculated using the formula below.

% Ash = (ash weight ÷ wet weight) × 100

#### 2.6.4. Lipid Analysis

Total lipid was extracted using the simplified chloroform:methanol method by Folch et al. (1957) [19] and AOAC official method 983.23 (2006) [20] at Texas Tech University. Approximately 1.0 g ± 0.02 g of sample was homogenized in 3.2 mL deionized water and 8.0 mL of chloroform and 8.0 mL methanol. Homogenized samples were placed on an orbital shaker at room temperature for 4 min. Then, 4 mL of deionized water was added, and the sample was placed on a shaker for an additional 2 min. Samples were centrifuged (Thermo Fisher Scientific, Waltham, MA, USA) for 10 min at 3500 rpm. The top layer of methanol solution was vacuumed off and 4 mL of the chloroform extract was pipetted into a pre-labeled, pre-weighed glass tube. Glass tubes were placed on a hot plate (Corning Incorporated, Corning, NY, USA) on a medium heat setting until chloroform was completely evaporated, leaving the residual lipid in the tube. Evaporated samples were dried in the oven for 20 min, cooled to room temperature and weighed. Lipid content was calculated using the formula below.

% Fat = (g residue ÷ g wet sample) × 2 × 100

#### 2.6.5. Fatty Acid Analysis

Fatty acid methyl esters (FAME) were separated using the gas chromatography, direct FAME synthesis method described by O’Fallon (2007) [21] at Texas Tech University. Approximately 1.0 g ± 0.02 g of homogenized sample was weighed into pre-washed, labeled, and weighed glass Pyrex tubes. Then, 1 mL of C:13 standard and 0.7 mL of 10 N KOH were added to each glass tube, and samples were vortexed. Following vortexing, 5.3 mL of methanol was added, and tubes were placed in a water bath at 55 °C for 1.5 h. The samples were shaken every 20 min. Tubes were then cooled to room temperature in a cold tap water bath, 0.58 mL of 24 N sulfuric acid was added and the samples were incubated in the water bath for another 1.5 h. Once the tubes were cooled to room temperature, 3.0 mL of hexane was added and vortexed for 5 min before being placed in the centrifuge for 5 min at 3500 rpm. For lean samples, 150 µL of the FAME hexane layer was diluted with 150 µL of hexane in an amber split insert gas chromatography vial. For fat samples 50 µL of the FAME hexane layer was diluted with 250 µL of hexane in an amber split insert gas chromatography vial.

Separation of FAME was performed using an Agilent Technologies (Santa Clara, CA, USA) 6890N series gas chromatography system with helium as the carrier gas. An HP-88 capillary column (100 m × 0.25 mm i.d.; Agilent Technologies, Santa Clara, CA, USA) was used in combination with a flame ionization detector. The oven was set at 140 °C for 5 min, then gradually increased to 240 °C at 4 °C per min. Samples were injected at a split ratio of 50:1. Fatty acid methyl esters were quantified by standards and internal standard calibration (Supelco 37 Component FAME Mix, Sigma-Aldrich, St. Louis, MO, USA).

#### 2.6.6. Cholesterol Analysis

Cholesterol analysis was determined using the cholesterol determination protocol AOAC 994.10 [22]. Approximately 1.0 g, recorded to the nearest 0.0001 g, was weighed into a boiling flask, and 2.0 mL of 50% KOH and 10.0 mL of 95% ethanol were added. The sample and solution were boiled and cooled to room temperature. Then, 10 mL of toluene was added, and the boiling flask was shaken and transferred to a separatory funnel. The 10 mL of 1.0 N aqueous KOH was added and the bottom, aqueous layer was discarded. The previous step was repeated with 5.0 mL of 0.5 N aqueous KOH. The toluene layer was washed 4 times by adding 5.0 mL of deionized water and discarding the aqueous layer. The final toluene layer was transferred to a 50 mL glass test tube that contained approximately 3.0 g of anhydrous Na_2_SO_4_. To prepare the samples for the gas chromatographer, 0.5 mL of the clear toluene layer was combined with 0.5 mL of standard solution and placed in a 2 mL vial. Samples were read on the GC mass spectrometer using an Agilent Technologies capillary column 122-4732E (Santa Clara, CA, USA) with Helium as the carrier gas. The oven temperature was set at 250 °C and the samples were injected at a split ratio of 20:1.

#### 2.6.7. Mineral Analysis

Minerals (Ca, Mg, K, Na, Fe, Zn, Cu, Mn, P, Se) were analyzed at Texas Tech University using the USDA wet ash procedure and AOAC official methods 985.35 [23]. Approximately 5.0 g, to the nearest 0.02 g, was weighed into an acid-washed, dried, and pre-weighed crucible. Samples were dried in the oven at 100 °C for 24 h and then ashed in the Thermolyne box furnace at 550 °C for 48 h. Ash samples were further digested by adding 3 mL deionized water and 3 mL 15.6 N nitric acid. Crucibles were slow-dried on a hot plate and re-ashed in the furnace at 550 °C for another 24 h. Then, 10 mL of 20% nitric acid was added to the ash sample. After standing for at least 5 h, the ash solution was filtered through ashless filter paper, transferred to a 50 mL volumetric flask, and brought to volume with deionized water. Ca and Mg samples were prepared and aspirated with lanthanum chloride solution. The remaining minerals, K, Na, Fe, Zn, Cu, and Mn, were aspirated directly. All samples and standards were aspirated on the atomic absorber (Shimadzu Scientific Instruments, Columbia, MD, USA) following the manufacturer’s instructions for wavelength.

Phosphorus and selenium were determined by Food Safety Net Services (San Antonio, TX, USA) using inductively coupled plasma mass spectrometry referencing AOAC methods 985.01, 999.10, and 993.14 [24,25,26].

#### 2.6.8. Vitamin E

Vitamin E content was measured by Food Safety Net Services (San Antonio, TX, USA) using high-performance liquid chromatography (HPLC) with a normal phase column, ultraviolet (UV) detection with external calibration, and standard recovery post-analysis.

#### 2.6.9. Vitamin D

Vitamin D analysis was performed by Food Safety Net Services (San Antonio, TX, USA) using LC-MS/MS (Liquid Chromatography with tandem triple quadrupole Mass Spectrometers).

#### 2.6.10. Vitamin A

Vitamin A content was measured by Food Safety Net Services (San Antonio, TX, USA) using the methods HPLC methods referenced from 11.1.1. AOAC methods 2001.13 and 2016.13 [27,28].

#### 2.6.11. Vitamin K

Vitamin K analysis was performed by Food Safety Net Services (San Antonio, TX, USA) using AOAC official method 999.15 [29] including HPLC and fluorescence detection.

#### 2.6.12. Amino Acid Analysis

Amino acid analysis was conducted by Food Safety Net Services (San Antonio, TX, USA) using the HPLC amino acid analysis protocol. A single sample for each cut and cook type was sent to the external lab; therefore, there is no measure of variance between replicates.

### 2.7. Total Caloric Value

The total caloric value of each USDA Prime beef cut was calculated using a base value of four kilocalories per gram of protein and nine kilocalories per gram of lipid for 100 g of the total sample. Caloric value was calculated for 100 g of raw sample and 100 g of cooked beef sample. Beef samples did not contain carbohydrates, so they were not included in the calculations.

### 2.8. Statistical Analysis

Data repeatability and precision were determined using blind duplicates and % coefficient of variation, with a threshold set at 5%. Accuracy was assessed by comparing measured values to those of the National Institute of Standards and Technology (NIST) Meat Homogenate 1546a certified nutrient values. Two main purposes of statistical analysis in this study included summarizing nutrient values for cuts and identifying differences in nutrient values among cuts. Estimated marginal means and pooled standard error of the mean (SEM) were determined for each nutrient, using R statistical software, version 1.4.1106 (R Studio, 2021). A linear model was fit for each cut type using the lm() function from base R, with cook type as the fixed effect. An analysis of variance (ANOVA) was conducted to determine if there was significance for the main effect of cut type and to generate a standard error of the mean. Significance level was set at α = 0.05 for all analyses. A pairwise comparison was used on significant ANOVA for mean separation and to determine where differences existed. Statistical analyses per nutrient were conducted using three composited samples (n = 3) for both raw and cooked beef cuts of strip loin steaks, tenderloin steaks, top sirloin steaks, ribeye steaks, and rib roasts. Three composites of each raw and cooked composited fat sample were also used to analyze the estimated marginal mean and pooled SEM of each nutrient. All statistical analyses were conducted on raw and cooked products separately; however, fat was compared between raw and cooked estimated means.

## 3. Results and Discussion

### 3.1. Separable Components

Separable components of lean, external fat, seam fat, and refuse are presented as a percentage of initial cut weight, as two steaks or one roast, for all cuts in Table 1. The average serving size for beef is 3 ounces, or 85 g, of cooked beef. Based on the weight of the separable lean portion of cooked cuts, the portion most commonly consumed, each unit (2 steaks or 1 roast) provided anywhere from about 4–10 servings (~300 to 830 g). Serving size ranges based on initial cut weight reveal the possibility of multiple servings of beef from one package of steaks (2 steaks) or one roast. Closely trimmed cuts, tenderloin steaks, and top sirloin steaks, possessed the greatest percent separable lean. Rib roasts contained the greatest percent external and seam fat, compared to other cuts. Results for separable component portions revealed slight differences when compared to USDA Choice and Select dissection data from the Nutrient Database Improvement Project [15,30]. Compared to raw USDA Choice and Select strip loin steak, tenderloin steak, ribeye steak, and rib roast, similar USDA Prime cuts, had a lesser percent separable lean, likely because of a greater percent external fat [15,30].

### 3.2. Nutrient Analysis of Separable Lean

Proximate values for total protein, lipid, moisture, and ash, as well as cholesterol, vitamin, and mineral content, for separable lean portions of raw and cooked cuts are listed in Table 2 and Table 3, respectively. Total percent lipid was significantly greater (*p* ≤ 0.01) in the raw and cooked ribeye and strip loin steak, compared to the tenderloin and top sirloin steak. Cooked, separable lean portions of top sirloin steak contained the least lipid percentage at 9.2%, while the cooked rib roast contained the greatest percentage of lipid content at 17.0%. Separable lean portions of sirloin steak contained the greatest numerical amount of cholesterol, 58.9 milligrams per 100 g of sample on a raw basis, but no significant difference was detected in cholesterol content between cuts for raw (*p* ≥ 0.44) or cooked (*p* ≥ 0.34) separable lean portions. Percent protein was inversely related to lipid percent, likely due to displaced protein for lipids, and the sirloin steak had the greatest percent protein while the rib roast had the least. Vitamins A, E, and D were below the standard level of detection for nearly all cuts. Vitamin K_1_ was the only fat-soluble vitamin detected consistently across all cuts, but at minimal levels compared to the established daily recommendation of 120 µg per day. Water soluble B vitamins play numerous critical roles in human health and all USDA Prime beef cuts contained thiamin, riboflavin, niacin, pantothenic acid, vitamin B6, and vitamin B12. In addition, all beef cuts contained a variety of minerals important for health, including potassium, phosphorus, sodium, magnesium, calcium, zinc, iron, copper, manganese, and selenium.

### 3.3. Nutrient Analysis of Composited Fat Samples

Predicted average values for lipid percentages were 70.9% and 70.3% for raw and cooked fat, respectively, revealing a majority of cook loss was due to a loss of moisture, shown by similar lipid portions. While there was not a detectable difference in percent lipid (*p* = 0.48), percent moisture was greater in the raw versus cooked composited fat samples (*p* ≤ 0.01). Inversely, protein percentage was greater in cooked versus raw fat (*p* = 0.01) at 8.62 and 5.94%, respectively. While many consumers assume the fat found in animal-source proteins is completely comprised of lipids, data show that visual fat is only about 70% lipid, compositionally, the remaining 30% is moisture and protein. Nutrient data for raw and cooked beef fat, and composited seam and external fat from all cuts, are listed in Table 4.

### 3.4. Nutrient Labeling Claims

Percent Daily Value (DV) was calculated using nutrient values per 100 g of raw separable lean from each beef cut and pre-established recommended intake levels for each nutrient. U.S. Food and Drug Administration (FDA) has established a daily value for vitamins, minerals, and macronutrients to relate the quantity of a nutrient within a serving of food to the human body’s requirement for that nutrient [31]. As presented in Table 5, all cuts were an excellent source, providing 20% or more of daily value, of protein, niacin, vitamin B12, selenium, and zinc. Additionally, strip steaks and ribeye steaks or roasts were a good source, providing 10 to 19% daily value, of thiamin, riboflavin, vitamin B6, pantothenic acid, and phosphorus. Sirloin steaks offer a good source of thiamin, riboflavin, vitamin B6, and phosphorus. No Prime beef cuts qualified as a good or excellent source of iron. Iron daily values are set at an elevated level to meet the increased needs for women versus men, so while Prime beef is characterized as an excellent source for men, cuts did not meet the requirement as a good or excellent source for women. Nonetheless, approximately half of iron in beef is heme-iron, the more digestible form of iron, and beef remains one of the best sources of dietary iron. Nutrition labeling claims suggest all prime cuts are nutrient-dense with essential vitamins and minerals.

### 3.5. Fatty Acid Profile

**Figure 2:**
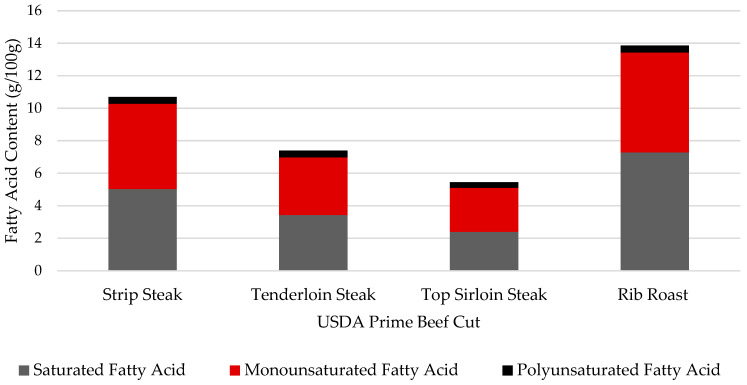
Breakdown of fatty acid composition by level of saturation for four raw, separable lean portions of USDA Prime beef cuts.

**Figure 3:**
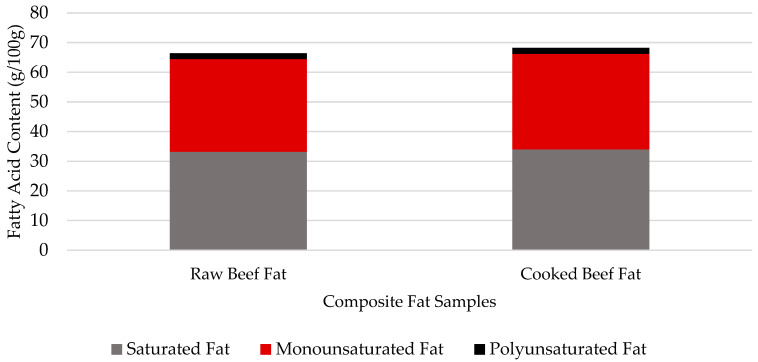
Breakdown of fatty acid composition by level of saturation for raw and cooked composited fat samples from five USDA Prime beef cuts.

Fatty acid concentrations for composited fat samples, as well as raw and cooked separable lean portions of USDA Prime beef cuts, are listed in Table 6, Table 7 and Table 8. Similar to the findings of Hunt et. al. (2016) [32], oleic acid (C18:1), palmitic acid (C16:0), and stearic acid (C18:0) were the most abundant fatty acids in all cuts and composited fat samples. Oleic acid is a heart-healthy monounsaturated fatty acid and stearic acid is known to have a neutral effect on cardiometabolic health, indicating two of the top three fatty acids found in beef may be neutral, or even beneficial for human health [33]. Saturated fatty acids composed the largest fraction of total fatty acids in composited fat samples while monounsaturated fatty acids made up the greatest proportion of total fatty acids in lean samples. Distribution of saturated, monounsaturated, and polyunsaturated fats is critical to understanding the nutritional value, as well as the benefits and implications of consuming beef. The breakdown of fatty acid composition is shown for raw, separable lean portions of USDA Prime beef cuts, as well as composited fat samples in Figure 2 and Figure 3 Composited fat samples, comprised of external and seam fat, were chemically more saturated, as presented in Table 6. Since the 1970s, meat processing has evolved to trimming excess fat from the external portions of beef cuts [34]. Furthermore, external and seam fat are often trimmed and discarded by the consumer, leaving separable lean as the most predominantly consumed portion of beef cuts. Separable lean portions of beef cuts have a chemical composition with a greater abundance of monounsaturated fatty acids compared to that of other portions. Monounsaturated and polyunsaturated fatty acids are often deemed “healthy fats” by the American Heart Association, as consumption of these fats can lead to a reduction in the concentration of low-density lipoprotein (LDL) in the blood with maintenance or an increase in high-density lipoproteins (HDL). A digital American Heart Association publication highlighted the benefits of consuming foods with monounsaturated fatty acids for decreasing risk factors of heart disease and stroke [35]. Monounsaturated fatty acids comprise approximately half of the total fatty acids and oleic acid (18:1n9) was the most abundant fatty acid in the analyzed beef samples, indicating USDA Prime beef can be beneficial in offering “heart-healthy fats” to be incorporated in a well-balanced diet. Ribeye steak and rib roast had the greatest amount of saturated fatty acids while the top sirloin and tenderloin steaks contained the least amount of saturated fatty acids. The 2020–2025 Dietary Guidelines for Americans recommend that 10% or fewer daily calories come from saturated fat. Based on a 2000-calorie diet, 100 g of raw USDA Prime strip loin steak, tenderloin steak, top sirloin steak, and rib roast only provide 2.3%, 1.5%, 1.1%, and 3.3%, respectively, of daily calorie intake in the form saturated fat. While total fatty acid content in USDA Prime beef is comprised of about 3 to 6.5% polyunsaturated fatty acids, the cuts analyzed were neither a good nor excellent source of PUFA. The USDA has established a lean meat definition as a single serving, 100 g raw or 85 g cooked, which contains less than 10 g total fat, less than 4.5 g saturated fat, and less than or equal to 95 mg cholesterol. According to the fatty acid and cholesterol analysis of the four raw, USDA Prime beef cuts, separable lean portions of tenderloin and top sirloin steak meet the qualifications for lean beef.

### 3.6. Amino Acid Profile

A single sample for each cut on a raw and cooked basis was sent to the external lab; therefore, there is no measure of variance between replicates. For these reasons, the standard error of the mean and *p*-value were not calculated. Numerical values for all essential and non-essential amino acids, as shown in Table 9 and Table 10, were similar to those reported for composite beef samples in FoodData Central [36]. The most prominent amino acids across all USDA Prime beef cuts, raw and cooked, were glutamic acid, aspartic acid, lysine, and leucine. While essential amino acids are well known for their role in muscle protein synthesis and maintenance of muscle health, recent research points to the role that non-essential amino acids may also play in muscle function [37]. Beef is a balanced source of both essential and non-essential amino acids.

### 3.7. Comparison to USDA Choice Beef Cuts

Nutrient values of beef cuts, raw separable lean portions only, of USDA Prime beef cuts were compared to separable lean nutrient data of USDA Choice beef cuts. Nutrient information for Choice cuts was derived from the USDA Food Data Central and is listed in Table 11. Compared to Choice beef, Prime cuts had a greater lipid percentage, and as a result, a lesser amount of minerals and water-soluble vitamins. All Choice cuts contained a greater amount of cholesterol; however, because cholesterol is largely found in the cell membrane within muscle fibers, previous research has indicated cholesterol does not vary greatly between USDA Grades [38]. Furthermore, prime beef contained a greater amount of “heart-healthy” monounsaturated fatty acids, particularly oleic acid.

### 3.8. Total Caloric Valuee

The total caloric value per 100 g for raw and 85 g for cooked, or one serving size, of each USDA Prime beef cut, is listed in Table 12. The total energy provided by all cooked USDA Prime beef cuts was greater than that of raw cuts because of a decrease in moisture, resulting in an increase in the concentration of protein and lipids. Ribeye steaks and rib roast provided the greatest number of calories while the tenderloin steak had the least number of calories.

### 3.9. Nutrient Loss and Retention Calculations

Nutrient loss and retention are reported for five USDA Prime beef cuts in Table 13. Cooked cuts were adjusted by percent weight loss, which is also listed in Table 11 as an average percent weight loss for each of the five beef cuts. As a result of the cooking process, there was a decrease in percent moisture, which accounts for a majority of cook loss by weight. Numerous water-soluble vitamins and minerals were also present in lower quantities in cooked samples, likely because of moisture loss. Protein and selenium are the only two nutrients that were consistently greater in the cooked versus raw samples.

## 4. Conclusions

Beef cuts derived from USDA Prime beef carcasses have a greater percentage of intramuscular fat, or marbling, resulting in a greater percent lipid in the separable lean portion compared to USDA Choice and Select beef cuts. Ruminant animals, like beef cattle, naturally deposit saturated fat; however, monounsaturated fatty acids comprised approximately half of the fatty acid content in USDA Prime beef cuts. Chemically, seam and external fat had a greater level of saturation, while lean portions of beef cuts contained a greater amount of monounsaturated fats. All USDA Prime beef cuts: strip loin steak, tenderloin steak, top sirloin steak, ribeye steak, and rib roast, provided a good or excellent source of protein and under-consumed, essential micronutrients. Separable lean portions of top sirloin steak and tenderloin steak met criteria set by USDA to be considered lean beef, containing less than 10 g total fat, 4.5 g or less of saturated fat, and less than 95 milligrams of cholesterol per 100 g. USDA Prime strip loin steaks, tenderloin steaks, top sirloin steaks, ribeye steaks, and rib roasts were determined to be a complete protein source rich in vitamin B12, niacin, zinc, selenium, and numerous other micronutrients, as well as heart-healthy monounsaturated fats.

## Figures and Tables

**Table 1:** Estimated marginal means and SEM ^1^ of initial cut weight and dissection components of raw and cooked USDA Prime beef cuts, expressed as a percent of initial cut weight.

Item	Initial Cut Weight ^2^ (g)	Separable Lean ^3^ (%)	External Fat ^4^ (%)	Seam Fat ^5^ (%)	Refuse ^6^ (%)
Raw					
Strip Loin Steak	752.0 ± 17.09	74.9 ± 4.64	12.2 ± 1.06	4.0 ± 0.77	2.7 ± 0.59
Tenderloin Steak	461.4 ± 38.15	95.9 ± 1.04	2.3 ± 0.56	1.3 ± 0.50	-
Top Sirloin Steak	583.1 ± 49.17	95.3 ± 0.82	3.6 ± 0.81	0.5 ± 0.28	-
Ribeye Steak	904.7 ± 45.39	74.9 ± 2.09	11.2 ± 1.07	13.1 ± 1.26	-
Rib Roast	1543.6 ± 78.23	71.6 ± 1.62	15.7 ± 1.68	12.2 ± 1.09	-
Cooked					
Strip Loin Steak	543.4 ± 22.14	81.7 ± 0.85	12.2 ± 0.77	1.8 ± 0.36	3.7 ± 0.60
Tenderloin Steak	313.1 ± 22.22	96.9 ± 1.03	0.9 ± 0.36	1.6 ± 0.53	0.4 ± 0.38
Top Sirloin Steak	419.1 ± 33.47	97.0 ± 0.65	1.9 ± 0.43	0.4 ± 0.17	0.5 ± 0.22
Ribeye Steak	616.2 ± 20.35	76.8 ± 1.22	11.2 ± 0.94	11.2± 0.97	0.2 ± 0.13
Rib Roast	1130.2 ± 66.71	73.7 ± 1.72	13.2 ± 1.16	12.3 ± 1.44	0.1 ± 0.12

^1^ Pooled (largest) standard error of the mean. ^2^ Initial cut weight for strip loin, tenderloin, ribeye, and top sirloin consisted of two steaks, and initial cut weight for rib roast consisted of one roast. ^3^ Separable lean included all lean, edible muscle tissue, intramuscular fat, and light, edible connective tissue. ^4^ External fat consisted of all adipose tissue around the external edges of the lean tissue. ^5^ Seam fat included all adipose tissue oriented between lean tissue. ^6^ Refuse was defined by heavy, inedible connective tissue and bone.

**Table 2:** Estimated marginal means and SEM ^1^ of proximate values as well as cholesterol, vitamin, and mineral content of raw, separable lean ^2^ components of four ^3^ USDA Prime beef cuts.

Item, Units	Strip Loin Steak	Tenderloin Steak	Top Sirloin Steak	Rib Roast ^4^	SEM ^5^	*p*-Value
Proximates						
Protein, %	21.7	21.8	23.2	21.9	0.484	0.17
Lipid, %	11.6 ^a^	9.2 ^b^	6.5 ^c^	12.7 ^a^	0.48	<0.01
Moisture, %	65.9 ^b^	69.2 ^a^	70.1 ^a^	64.6 ^b^	0.39	<0.01
Ash, %	0.9 ^b^	1.0 ^ab^	1.1 ^a^	0.8 ^c^	0.03	<0.01
Cholesterol, mg/100 g	55.4	57.2	58.9	58.1	1.51	0.44
Vitamins						
Vitamin A (total), IU/100 g ^6^	0.33	*	*	*	16.7	-
Vitamin D (total D2 and D3), IU/100 g ^7^	*	*	*	*	-	-
Vitamin E, IU/kg ^8^	*	*	*	*	-	-
Vitamin K_1_, µg/100 g	0.93	0.50	0.13	0.83	0.314	0.33
Vitamin B_1_ (Thiamin), mg/100 g	0.08	0.14	0.12	0.15	0.021	0.23
Vitamin B_2_ (Riboflavin), mg/100 g	0.22	0.18	0.13	0.11	0.068	0.65
Vitamin B_3_ (Niacin), mg/100 g	4.89	5.85	4.42	4.37	0.753	0.51
Vitamin B_5_ (Pantothenic Acid), mg/100 g	0.42	0.55	0.57	0.53	0.067	0.42
Vitamin B_6_ (Pyridoxine), µg/100 g	0.10 ^b^	0.26 ^a^	0.20 ^ab^	0.32 ^a^	0.027	<0.01
Vitamin B_12_ (Cyanocobalamin), µg/100 g	1.48	1.51	1.76	1.69	0.181	0.65
Minerals						
Potassium, mg/100 g	289 ^a^	322 ^ab^	340 ^a^	272 ^b^	14.5	0.04
Phosphorus, mg/100 g	186 ^bc^	203 ^ab^	219 ^a^	170 ^c^	4.87	<0.01
Sodium, mg/100 g	63.4 ^ab^	64.5 ^a^	62.5 ^ab^	60.1 ^b^	0.86	0.03
Magnesium, mg/100 g	21.8 ^c^	22.8 ^b^	23.7 ^a^	20.3 ^d^	0.167	<0.01
Calcium, mg/100 g	2.79	2.57	3.72	2.42	0.372	0.14
Zinc, mg/100 g	2.30 ^b^	2.18 ^b^	2.47 ^ab^	2.68 ^a^	0.076	<0.01
Iron, mg/100 g	1.31	1.46	1.57	1.39	0.139	0.63
Copper, mg/100 g	0.10 ^b^	0.15 ^a^	0.14 ^ab^	0.12 ^ab^	0.010	0.03
Manganese, mg/100 g	≤0.02	≤0.02	≤0.02	≤0.02	-	-
Selenium, µg/100 g	20.0	22.3	22.2	22.4	1.29	0.38

^1^ Pooled (largest) standard error of the mean. ^2^ Separable lean included all lean, edible muscle tissue, intramuscular fat, and light, edible connective tissue. ^3^ Strip loin steaks, top sirloin steaks, tenderloin steaks, and rib roasts were analyzed for all nutrients. ^4^ Ribeye steaks and rib roasts were combined for raw analysis because of the same origin. ^5^ Pooled standard error of least squared means. ^6^ Cuts denoted with a “*” were below the detectable limit of 50 IU/100 g for total Vitamin A. ^7^ Cuts denoted with a “*” were below the detectable limit of 8 IU/100 g for total Vitamin D. ^8^ Cuts denoted with a “*” were below the detectable limit of 1 IU/kg for Vitamin E. ^a–d^ Estimated marginal means, within a row, with different superscripts differ (*p* < 0.05).

**Table 3:** Estimated marginal means and SEM ^1^ of proximate values as well as cholesterol, vitamin, and mineral content of cooked, separable lean ^2^ components of five ^3^ USDA Prime beef cuts.

Item, Units	Strip Loin Steak	Tenderloin Steak	Top Sirloin Steak	Ribeye Steak	Rib Roast	SEM ^4^	*p*-Value
Proximates							
* *Protein, %	30.7 ^ab^	30.9 ^ab^	33.1 ^a^	30.2 ^ab^	29.2 ^b^	0.68	0.03
* *Lipid, %	14.0 ^ab^	12.1 ^bc^	9.2 ^c^	16.6 ^a^	17.0 ^a^	0.89	<0.01
* *Moisture, %	54.7 ^ab^	57.2 ^ab^	58.5 ^a^	53.2 ^b^	53.8 ^b^	0.87	<0.01
* *Ash, %	1.0 ^b^	1.3 ^a^	1.3 ^a^	0.9 ^b^	1.0 ^b^	0.03	<0.01
* *Cholesterol, mg/100 g	77.4	82.9	85.8	81.7	76.7	3.37	0.34
Vitamins							
* *Vitamin A (total), IU/100 g ^5^	*	78.8	*	*	30.6	22.3	0.44
* *Vitamin D (total D2 and D3), IU/100 g ^6^	*	*	*	*	*	-	-
* *Vitamin E, IU/kg ^7^	*	*	*	*	*	-	-
* *Vitamin K1, µg/100 g	0.17 ^b^	2.27 ^a^	0.17 ^b^	0.47 ^ab^	1.07 ^ab^	0.445	0.04
* *Vitamin B_1_ (Thiamin), mg/100 g	0.13	0.11	0.13	0.13	0.17	0.026	0.64
* *Vitamin B_2_ (Riboflavin), mg/100 g	0.14	0.08	0.15	0.12	0.14	0.039	0.78
* *Vitamin B_3_ (Niacin), mg/100 g	6.64 ^a^	3.08 ^b^	6.38 ^a^	4.43 ^ab^	3.56 ^ab^	0.682	0.01
* *Vitamin B_5_ (Pantothenic Acid), mg/100 g	0.56	0.37	0.46	0.41	0.56	0.067	0.23
* *Vitamin B_6_ (Pyridoxine), µg/100 g	0.24	0.13	0.25	0.18	0.19	0.046	0.40
* *Vitamin B_12_ (Cyanocobalamin), µg/100 g	1.79	1.18	1.50	1.57	1.81	0.331	0.67
Minerals							
* *Potassium, mg/100 g	310	363	308	342	310	19.0	0.22
* *Phosphorus, mg/100 g	222 ^bc^	244 ^ab^	270 ^a^	213 ^bc^	207 ^c^	7.3	<0.01
* *Sodium, mg/100 g	66.2	72.4	69.9	71.4	68.3	2.78	0.56
* *Magnesium, mg/100 g	23.9 ^b^	26.1 ^a^	26.1 ^a^	22.2 ^bc^	21.3 ^c^	0.45	<0.01
* *Calcium, mg/100 g	3.95	2.93	3.87	3.17	3.02	0.409	0.30
* *Zinc, mg/100 g	2.75 ^ab^	2.48 ^b^	2.88 ^ab^	2.99 ^a^	2.96 ^a^	0.087	0.01
* *Iron, mg/100 g	1.42	1.84	2.06	1.44	1.47	0.175	0.09
* *Copper, mg/100 g	0.14	0.16	0.28	0.14	0.12	0.081	0.67
* *Manganese, mg/100 g	≤0.02	≤0.02	≤0.02	≤0.02	≤0.02	-	-
* *Selenium, µg/100 g	29.4	31.6	33.2	29.3	29.7	2.12	0.64

^1^ Pooled (largest) standard error of the mean. ^2^ Separable lean included all lean, edible muscle tissue, intramuscular fat, and light, edible connective tissue. ^3^ Strip loin steaks, top sirloin steaks, tenderloin steaks, ribeye steaks, and rib roasts were analyzed for all nutrients. ^4^ Pooled (largest) standard error of least squared means. ^5^ Cuts denoted with a “*” were below the detectable limit of 50 IU/100 g for total Vitamin A. ^6^ Cuts denoted with a “*” were below the detectable limit of 8 IU/100 g for total Vitamin D. ^7^ Cuts denoted with a “*” were below the detectable limit of 1 IU/kg for Vitamin E. ^a–c^ Estimated marginal means, within a row, with different superscripts differ (*p* < 0.05).

**Table 4:** Estimated marginal means and SEM ^1^ of proximate values, cholesterol content, and vitamin and mineral content of raw and cooked composited fat samples ^2^ from five USDA Prime beef cuts.

Item	Raw Beef Fat	Cooked Beef Fat	SEM	*p*-Value
Proximate Values				
Protein, %	5.94 ^b^	8.62 ^a^	0.341	0.01
Lipid, %	70.9	70.3	0.531	0.48
Moisture, %	23.0 ^a^	18.5 ^b^	0.532	<0.01
Ash, %	0.22 ^b^	0.48 ^a^	0.027	<0.01
Cholesterol, mg/g	70.5	80.1	1.95	0.03
Vitamins				
Vitamin A (total), IU/100 g ^3^	*	79.6	28.10	-
Vitamin D (total D2 and D3), IU/100 g ^4^	*	*	-	-
Vitamin E, IU/kg ^5^	*	*	-	-
Vitamin K1, µg/mg	0.40 ^b^	1.67 ^a^	0.655	0.02
Vitamin B1 (Thiamin), mg/100 g	0.12	0.11	0.029	0.82
Vitamin B2 (Riboflavin), mg/100 g	0.10	0.09	0.047	0.89
Vitamin B3 (Niacin), mg/100 g	4.93 ^a^	2.70 ^b^	0.518	0.04
Vitamin B5 (Pantothenic Acid), mg/100 g	0.47	0.49	0.161	0.91
Vitamin B6 (Pyridoxine), µg/100 g	0.22	0.15	0.075	0.55
Vitamin B12 (Cyanocobalamin), µg/100 g	1.40	1.21	0.332	0.71
Minerals				
Potassium, mg/100 g	132 ^b^	226 ^a^	18.1	0.02
Phosphorus, mg/100 g	54.9 ^b^	93.9 ^a^	1.50	<0.01
Sodium, mg/100 g	35.2 ^b^	49.8 ^a^	2.96	0.03
Magnesium, mg/100 g	6.31 ^b^	12.1 ^a^	0.562	<0.01
Calcium, mg/100 g	3.96	3.05	0.686	0.40
Zinc, mg/100 g	0.99 ^b^	1.39 ^a^	0.058	<0.01
Iron, mg/100 g	0.69 ^b^	1.16 ^a^	0.042	<0.01
Copper, mg/100 g	0.13	0.06	0.041	0.31
Manganese, mg/100 g	≤0.02	≤0.02	-	-
Selenium, µg/100 g	0.00	7.13	2.52	0.12

^1^ Pooled (largest) standard error of least squared means. ^2^ Seam (adipose tissue between lean tissue) and external (adipose tissue around the exterior edges of the cut) fat were dissected from USDA Prime strip loin steaks, top sirloin steaks, tenderloin steaks, ribeye steaks, and rib roasts. ^3^ Cuts denoted with a “*” were below the detectable limit of 50 IU/100 g for total Vitamin A. ^4^ Cuts denoted with a “*” were below the detectable limit of 8 IU/100 g for total Vitamin D. ^5^ Cuts denoted with a “*” were below the detectable limit of 1 IU/kg for Vitamin E. ^a,b^ Estimated marginal means, within a row, with different superscripts differ (*p* < 0.05).

**Table 5:** Percentage ^1^ of nutrient Daily Value contributed to by 100 g of raw separable lean tissue ^2^ from five ^3^ USDA Prime beef cuts and their qualification as a USDA “Good” ^4^ or “Excellent” ^5^ source for varying nutrients.

Nutrient	Daily Value	Strip Loin Steak	Tenderloin Steak	Top Sirloin Steak	Ribeye Steak
Protein	50 g	43.4 ^E^	43.6 ^E^	46.4 ^E^	43.8 ^E^
Thiamin (B1)	1.2 mg	6.7	11.7 ^G^	10.0 ^G^	12.5 ^G^
Riboflavin (B2)	1.3 µg	16.9 ^G^	13.8 ^G^	10.0 ^G^	8.5
Niacin (B3)	16 mg	30.6 ^E^	36.6 ^E^	27.6 ^E^	27.3 ^E^
Vitamin B6	1.7 mg	5.9	15.3 ^G^	11.8 ^G^	18.8 ^G^
Vitamin B12	2.4 µg	61.7 ^E^	62.9 ^E^	73.3 ^E^	70.4 ^E^
Selenium	55 µg	36.4 ^E^	42.4 ^E^	40.4 ^E^	40.7 ^E^
Phosphorus	1250 mg	14.9 ^G^	16.2 ^G^	17.5 ^G^	13.6 ^G^
Zinc	11 mg	20.9 ^E^	19.8 ^G^	22.5 ^E^	24.4 ^E^
Iron	18 mg	7.2	8.1	8.7	7.7

^1^ Percentages were calculated based on daily value according to the FDA reference guide for nutrition labeling. ^2^ Separable lean included all lean, edible muscle tissue, intramuscular fat, and light, edible connective tissue. ^3^ Strip loin steak, tenderloin steak, top sirloin steak, ribeye steak, and rib roast nutrients were calculated. ^4^ Providing between 10 and 19% of the daily value qualifies the item to be labeled as a “good source” of the nutrient. ^5^ Providing over 20% of the daily value qualifies the item to be labeled as an “excellent source” of the nutrient. ^E^ Item qualifies for extra labeling as an “Excellent source” of the nutrient. ^G^ Item qualifies for extra labeling as a “Good source” of the nutrient.

**Table 6:** Estimated marginal means and SEM ^1^ of fatty acid content per 100 g of raw and cooked composited seam and external fat samples ^2^ from five USDA Prime beef cuts.

Fatty Acid, g/100 g	Cooked Beef Fat	Raw Beef Fat	SEM	*p*-Value ^3^
Saturated Fatty Acids	34.03	33.22	0.841	0.53
10:0	0.03	0.03	0.002	0.60
12:0	0.05	0.05	0.003	0.39
14:0	2.33	2.32	0.086	0.95
15:0	0.37	0.36	0.024	0.89
16:0	18.92	18.4	0.516	0.51
17:0	1.00	0.96	0.053	0.63
18:0	11.16	10.93	0.212	0.49
19:0	0.03	0.03	<0.001	0.28
20:0	0.13	0.13	0.005	0.96
22:0	0.01	0.01	0.002	0.88
24:0	0.01	0.01	<0.001	0.42
Monounsaturated Fatty Acids	32.21	31.29	0.726	0.42
14:1*n*5	0.65	0.63	0.027	0.73
16:1 trans	0.05	0.06	0.009	0.54
16:1*n*7	2.04	1.95	0.047	0.25
17:1	0.01	0.01	0.001	0.59
18:1 trans	3.29	3.19	0.192	0.71
18:1*n*9	25.35	24.59	0.483	0.33
18:1*n*7	0.21	0.22	0.006	0.38
19:1	0.01	0.01	0.001	0.46
20:1*n*5	0.00	0.04	0.029	0.37
20:1*n*8	0.08	0.08	0.002	0.69
20:1*n*11	0.47	0.47	0.02	1.00
24:1*n*9	0.02	0.02	0.001	0.62
Polyunsaturated Fatty Acids	2.03	1.93	0.139	0.62
18:2 trans	0.08	0.08	0.039	0.97
18:3*n*6	0.18	0.18	0.004	0.93
18:2*n*6	1.56	1.46	0.101	0.51
20:2	0.03	0.03	0.001	0.24
20:3*n*6	0.06	0.06	0.002	0.62
20:3*n*3	0.01	0.00	0.001	0.41
20:4*n*6	0.04	0.03	0.003	0.21
22:4	0.02	0.02	0.001	0.62
22:5*n*3	0.04	0.05	0.006	0.68
22:6*n*3	0.02	0.02	0.007	0.66
*n*-3 Fatty Acids	0.07	0.08	0.008	0.60
*n*-6 Fatty Acids	1.83	1.72	0.105	0.50
*n*-6:*n*-3	26	22	--	--

^1^ Pooled (largest) standard error of least squared means. ^2^ Seam (adipose tissue between lean tissue) and external (adipose tissue around the exterior edges of the cut) fat were dissected from USDA Prime strip loin steaks, top sirloin steaks, tenderloin steaks, ribeye steaks, and rib roasts. ^3^ Observed significance level for cook type differences.

**Table 7:** Estimated marginal means and SEM ^1^ of fatty acid content per 100 g of raw, separable lean ^2^ portions of four ^3^ USDA Prime beef cuts.

Fatty Acid, g/100 g	Strip Loin Steak	Tenderloin Steak	Top Sirloin Steak	Rib Roast ^4^	SEM	*p*-Value
Saturated Fatty Acids	5.03 ^ab^	3.43 ^ab^	2.39 ^b^	7.28 ^a^	0.900	0.02
10:0	0.01 ^a^	0.00 ^b^	0.00 ^c^	0.01 ^a^	<0.001	<0.01
12:0	0.01 ^a^	0.01 ^b^	0.00 ^c^	0.01 ^a^	<0.001	<0.01
14:0	0.36 ^ab^	0.23 ^bc^	0.15 ^c^	0.45 ^a^	0.036	<0.01
15:0	0.07	0.05	0.04	0.08	0.011	0.14
16:0	3.07 ^ab^	1.97 ^ab^	1.40 ^b^	4.37 ^a^	0.536	0.02
17:0	0.13	0.09	0.06	0.21	0.033	0.07
18:0	1.38 ^ab^	1.05 ^ab^	0.72 ^b^	2.13 ^a^	0.288	0.04
20:0	0.01 ^bc^	0.02 ^ab^	0.01 ^c^	0.02 ^a^	0.001	<0.01
Monounsaturated Fatty Acids	5.25 ^a^	3.54 ^b^	2.71 ^b^	6.14 ^a^	0.342	<0.01
14:1*n*5	0.11 ^a^	0.06 ^b^	0.04 ^c^	0.10 ^a^	0.003	<0.01
16:1 trans	0.01	0.00	0.00	0.01	0.001	0.10
16:1*n*7	0.40 ^a^	0.21 ^b^	0.17 ^b^	0.38 ^a^	0.012	<0.01
17:1	0.04	0.07	0.05	0.04	0.023	0.75
18:1 trans	0.35 ^ab^	0.29 ^bc^	0.21 ^c^	0.46 ^a^	0.030	<0.01
18:1*n*9	4.19 ^ab^	2.74 ^bc^	2.10 ^c^	5.00 ^a^	0.337	<0.01
18:1*n*7	0.08	0.11	0.09	0.08	0.033	0.86
20:1*n*8	0.01	0.00	0.00	0.01	0.002	0.10
20:1*n*11	0.05 ^a^	0.05 ^a^	0.04 ^b^	0.07 ^a^	0.004	<0.01
Polyunsaturated Fatty Acids	0.42	0.43	0.35	0.43	0.023	0.09
18:2 trans	0.02	0.01	0.01	0.01	0.004	0.20
18:3*n*6	0.02 ^ab^	0.02 ^bc^	0.01 ^c^	0.03 ^a^	0.002	<0.01
18:2*n*6	0.30 ^ab^	0.31 ^a^	0.24 ^b^	0.30 ^ab^	0.015	0.04
20:2	0.00 ^ab^	0.00 ^ab^	0.00 ^b^	0.01 ^a^	<0.001	0.01
20:3*n*6	0.01	0.01	0.01	0.02	0.005	0.51
20:4*n*6	0.05 ^ab^	0.05 ^a^	0.05 ^ab^	0.04 ^b^	0.003	0.03
22:4	0.01	0.00	0.00	0.01	0.001	0.10
22:5*n*3	0.01	0.01	0.01	0.01	0.001	0.49
*n*-3 Fatty Acids	0.01	0.02	0.02	0.02	0.001	0.18
*n*-6 Fatty Acids	0.38	0.39	0.31	0.38	0.019	0.07
*n*-6:*n*-3	38	20	16	19	--	--

^1^ Pooled (largest) standard error of least squared means. ^2^ Separable lean included all lean, edible muscle tissue, intramuscular fat, and light, edible connective tissue. ^3^ Strip loin steaks, top sirloin steaks, tenderloin steaks, and rib roasts were analyzed for all nutrients. ^4^ Ribeye steaks and rib roasts were combined for raw analysis because of the same origin. ^a–c^ Estimated marginal means within a row with different superscripts differ (*p* < 0.05).

**Table 8:** Estimated marginal means and SEM ^1^ of fatty acid content in g per 100 g of cooked, separable lean ^2^ portions of five ^3^ USDA Prime beef cuts.

Fatty Acid, g/100 g	Strip Loin Steak	Tender-Loin Steak	Top Sirloin Steak	Ribeye	Rib Roast	SEM	*p*-Value
Saturated Fatty Acids	6.04 ^a^	5.74 ^ab^	3.55 ^b^	7.94 ^a^	7.34 ^a^	0.495	<0.01
10:0	0.01 ^ab^	0.01 ^ab^	0.00 ^b^	0.01 ^a^	0.01 ^a^	0.001	<0.01
12:0	0.01 ^ab^	0.02 ^a^	0.01 ^ab^	0.01 ^ab^	0.01 ^b^	0.001	0.04
14:0	0.43 ^ab^	0.36 ^bc^	0.21 ^c^	0.53 ^a^	0.52 ^ab^	0.035	<0.01
15:0	0.07 ^a^	0.08 ^a^	0.05 ^b^	0.09 ^a^	0.08 ^a^	0.004	<0.01
16:0	3.66 ^ab^	3.26 ^bc^	2.00 ^c^	4.69 ^a^	4.38 ^ab^	0.286	<0.01
17:0	0.16 ^ab^	0.16 ^ab^	0.10 ^b^	0.22 ^a^	0.19 ^a^	0.017	<0.01
18:0	1.67 ^ab^	1.82 ^ab^	1.14 ^b^	2.35 ^a^	2.11 ^a^	0.166	<0.01
19:0	0.01 ^a^	0.00 ^b^	0.00 ^b^	0.01 ^a^	0.01 ^a^	<0.001	<0.01
20:0	0.02	0.03	0.02	0.03	0.03	0.003	0.08
Monounsaturated Fatty Acids	6.42 ^ab^	5.10 ^bc^	4.04 ^c^	7.52 ^a^	7.64 ^a^	0.424	<0.01
13:1	0.01	0.00	0.00	0.01	0.00	0.004	0.57
14:1*n*5	0.12 ^a^	0.08 ^b^	0.05 ^b^	0.14 ^a^	0.14 ^a^	0.008	<0.01
16:1 trans	0.01	0.00	0.00	0.01	0.01	0.002	0.59
16:1*n*7	0.49 ^a^	0.30 ^b^	0.23 ^b^	0.51 ^a^	0.53 ^a^	0.032	<0.01
17:1	0.08	0.1	0.08	0.11	0.09	0.033	0.95
18:1 trans	0.46	0.45	0.34	0.58	0.59	0.063	0.09
18:1*n*9	5.02 ^ab^	3.92 ^bc^	3.14 ^c^	5.90 ^a^	5.99 ^a^	0.344	<0.01
18:1*n*7	0.14	0.16	0.14	0.17	0.17	0.046	0.97
20:1*n*8	0.01	0.00	0.00	0.01	0.01	0.003	0.44
20:1*n*11	0.07 ^bc^	0.07 ^bc^	0.05 ^c^	0.09 ^ab^	0.10 ^a^	0.006	<0.01
Polyunsaturated Fatty Acids	0.54	0.64	0.54	0.61	0.62	0.050	0.48
18:2 trans	0.02	0.02	0.01	0.02	0.03	0.004	0.20
18:3*n*6	0.03 ^ab^	0.02 ^ab^	0.02 ^b^	0.03 ^a^	0.03 ^a^	0.003	0.01
18:2*n*6	0.38	0.45	0.37	0.44	0.44	0.037	0.39
20:2	0.01	0.01	0.01	0.01	0.01	0.001	0.13
20:3*n*6	0.02	0.02	0.02	0.02	0.02	0.002	0.76
20:4*n*6	0.06 ^a^	0.08 ^a^	0.08 ^a^	0.05 ^a^	0.05 ^a^	0.005	0.03
22:4	0.01	0.01	0.01	0.01	0.01	0.001	0.30
22:5*n*3	0.01	0.02	0.02	0.01	0.01	0.002	0.60
*n*-3 Fatty Acids	0.02	0.03	0.03	0.02	0.02	0.003	0.36
*n*-6 Fatty Acids	0.49	0.58	0.48	0.55	0.55	0.044	0.50
*n*-6:*n*-3	25	19	16	28	28	--	--

^1^ Pooled (largest) standard error of least squared means. ^2^ Separable lean included all lean, edible muscle tissue, intramuscular fat, and light, edible connective tissue. ^3^ Strip loin steaks, top sirloin steaks, tenderloin steaks, ribeye steaks, and rib roasts were analyzed for all nutrients. ^a–c^ Estimated marginal means, within a row, with different superscripts differ (*p* < 0.05).

**Table 9:** Amino acid concentration per 100 g of separable lean ^1^ portions of four ^2^ raw, USDA Prime beef cuts.

Amino Acid, g/100 g	Strip Loin Steak	Tenderloin Steak	Top Sirloin Steak	Rib Roast ^3^
Essential				
Histidine	0.74	0.69	0.78	0.69
Isoleucine	1.00	1.03	1.00	0.97
Leucine	1.71	1.78	1.74	1.67
Lysine	1.83	1.89	1.85	1.79
Methionine	0.60	0.62	0.60	0.57
Phenylalanine	0.86	0.87	0.87	0.83
Threonine	0.95	0.98	0.96	0.92
Tryptophan	0.25	0.25	0.25	0.25
Valine	1.05	1.07	1.07	1.02
Non-Essential				
Alanine	1.36	1.25	1.27	1.27
Arginine	1.42	1.39	1.38	1.34
Aspartic Acid	1.95	1.97	1.95	1.88
Cystine	0.24	0.25	0.25	0.24
Glutamic Acid	3.17	3.21	3.16	3.05
Glycine	1.18	0.86	0.92	0.95
Proline	1.02	0.85	0.87	0.90
Serine	0.83	0.83	0.82	0.80
Tyrosine	0.72	0.75	0.75	0.71

^1^ Separable lean included all lean, edible muscle tissue, intramuscular fat, and light, edible connective tissue. ^2^ Strip loin steaks, top sirloin steaks, tenderloin steaks, and rib roasts were analyzed for all nutrients. ^3^ Ribeye steaks and rib roasts were combined for raw analysis because of the same origin.

**Table 10:** Amino acid concentration per 100 g of separable lean ^1^ portions of five ^2^ cooked, USDA Prime beef cuts.

Amino Acid, g/100 g	Strip Loin Steak	Tenderloin Steak	Top Sirloin Steak	Ribeye Steak	Rib Roast
Essential					
Histidine	0.98	0.93	1.05	0.91	0.87
Isoleucine	1.46	1.47	1.48	1.40	1.32
Leucine	2.46	2.56	2.58	2.41	2.25
Lysine	2.67	2.72	2.76	2.61	2.44
Methionine	0.84	0.88	0.88	0.82	0.77
Phenylalanine	1.21	1.25	1.29	1.20	1.13
Threonine	1.38	1.40	1.43	1.33	1.25
Tryptophan	0.25	0.25	0.25	0.25	0.25
Valine	1.51	1.53	1.56	1.45	1.37
Non-Essential					
Alanine	1.82	1.81	1.88	1.79	1.76
Arginine	1.96	1.95	2.02	1.96	1.80
Aspartic Acid	2.79	2.86	2.92	2.76	2.59
Cystine	0.34	0.34	0.34	0.33	0.30
Glutamic Acid	4.45	4.62	4.62	4.38	4.18
Glycine	1.37	1.26	1.45	1.40	1.52
Proline	1.28	1.24	1.34	1.30	1.34
Serine	1.17	1.18	1.22	1.14	1.10
Tyrosine	1.08	1.09	1.11	1.05	0.97

^1^ Separable lean included all lean, edible muscle tissue, intramuscular fat, and light, edible connective tissue. ^2^ Strip loin steaks, top sirloin steaks, tenderloin steaks, ribeye steaks, and rib roasts were analyzed for all nutrients.

**Table 11:** Nutrient values of four ^1^ raw USDA Prime beef cuts, separable lean ^2^ only, compared to separable lean nutrient data of the same four USDA Choice cuts as listed in the USDA National Nutrient Database for Standard Reference.

Item, Unit	USDA Prime Nutrient Data	USDA Choice Nutrient Data
Strip Steak ^3^		
Protein, %	21.7	22.9
Total Lipid, %	11.6	6.34
Zinc, mg/100 g	2.3	3.8
Iron, mg/100 g	1.3	1.9
Niacin, mg/100 g	4.9	7.0
Vitamin B12, µg/100 g	1.5	1.7
SFA, g/100 g	5.03	2.52
MUFA, g/100 g	5.25	2.95
Oleic Acid, C18:1n9	4.19	2.36
PUFA, g/100 g	0.42	0.39
Cholesterol, mg/100 g	55	58
Tenderloin Steak ^4^		
Protein, %	21.8	21.8
Total Lipid, %	9.2	6.2
Zinc, mg/100 g	2.4	3.3
Iron, mg/100 g	1.4	2.6
Niacin, mg/100 g	5.9	4.5
Vitamin B12, µg/100 g	1.5	3.4
SFA, g/100 g	3.43	2.12
Oleic Acid, C18:1n9	2.74	1.85
MUFA, g/100 g	3.54	2.34
PUFA, g/100 g	0.43	0.45
Cholesterol, mg/100 g	57	60
Ribeye Steak ^5^		
Protein, %	21.9	21.4
Total Lipid, %	12.7	8.5
Zinc, mg/100 g	2.7	5.6
Iron, mg/100 g	1.6	2.0
Niacin, mg/100 g	4.4	5.5
Vitamin B12, µg/100 g	1.7	2.0
SFA, g/100 g	7.28	3.06
MUFA, g/100 g	6.14	3.58
Oleic Acid, C18:1n9	5.00	2.86
PUFA, g/100 g	0.43	0.47
Cholesterol, mg/100 g	58	63
Top Sirloin Steak ^6^		
Protein, %	23.2	21.9
Total Lipid, %	6.5	4.6
Zinc, mg/100 g	2.5	4.1
Iron, mg/100 g	1.5	1.6
Niacin, mg/100 g	4.4	7.4
Vitamin B12, µg/100 g	1.8	1.2
SFA, g/100 g	2.39	1.71
Oleic Acid, C18:1n9	2.10	1.72
MUFA, g/100 g	2.71	1.86
PUFA, g/100 g	0.35	0.21
Cholesterol, mg/100 g	59	61

^1^ Beef cuts were strip loin steak, top sirloin steak, tenderloin steak, ribeye steak, and rib roast. ^2^ Separable lean included all lean, edible muscle tissue, intramuscular fat, and light, edible connective tissue. ^3^ USDA National Nutrient Database for SR Legacy #23370. ^4^ USDA National Nutrient Database for SR Legacy #23373. ^5^ USDA National Nutrient Database for SR Legacy #23177. ^6^ USDA National Nutrient Database for SR Legacy #23625.

**Table 12:** Total caloric value ^1^ of 100 g of raw and 85 g of cooked, separable lean ^2^ portions of USDA Prime beef cuts.

Item	Total Caloric Content
Raw (kcal/100 g sample)	
Strip Loin Steak	191.2
Tenderloin Steak	170.0
Top Sirloin Steak	151.3
Rib Roast	201.9
Cooked (kcal/85 g sample)	
Strip Loin Steak	211.8
Tenderloin Steak	197.6
Top Sirloin Steak	182.9
Ribeye Steak	229.7
Rib Roast	229.3

^1^ Caloric value calculated using 4 kcal/g protein and 9 kcal/g lipid. Carbohydrates were not present in beef cuts, so were not accounted for in calculations. ^2^ Separable lean included all lean, edible muscle tissue, intramuscular fat, and light, edible connective tissue.

**Table 13:** Nutrient loss and retention after cooking ^1^ shown by nutrient value differences between cooked and raw separable lean portions of USDA Prime beef cuts adjusted ^2^ by percent weight loss during cooking.

Item	Strip Loin Steak	Tenderloin Steak	Top Sirloin Steak	Ribeye Steak	Rib Roast
Percent Cook Loss ± SEM	23.6± 0.93	24.8 ± 0.91	25.3 ± 0.91	23.9 ± 0.93	25.4 ± 0.91
Proximates					
Protein, %	1.8	1.4	1.5	1.1	−0.1
Lipid, %	−0.9	−0.1	0.4	−0.1	0.0
Moisture, %	−24.1	−26.2	−26.4	−24.1	−24.5
Ash, %	−0.1	0.0	−0.1	−0.1	−0.1
Cholesterol, mg/100 g	3.7	5.1	5.2	4.1	−0.9
Vitamins					
Vitamin A (total), IU/100 g	−0.3	59.3	0.0	0.0	22.8
Vitamin D (total D2 and D3), IU/100 g	--	--	--	--	--
Vitamin E, IU/kg	--	--	--	--	--
Vitamin K1, µg/100 g	−0.8	1.2	--	−0.5	--
Vitamin B1 (Thiamin), mg/100 g	--	−0.1	--	−0.1	--
Vitamin B2 (Riboflavin), mg/100 g	−0.1	−0.1	--	--	--
Vitamin B3 (Niacin), mg/100 g	0.2	−3.5	0.3	−1.0	−1.7
Vitamin B5 (Pantothenic Acid), mg/100 g	--	−0.3	−0.2	−0.2	−0.1
Vitamin B6 (Pyridoxine), mg/100 g	0.1	−0.2	--	−0.2	−0.2
Vitamin B12 (Cyanocobalamin), µg/100 g	−0.1	−0.6	−0.6	−0.5	−0.3
Minerals					
Potassium, mg/100 g	−52.2	−49.0	−109.9	−11.7	−40.7
Phosphorus, mg/100 g	−16.4	−19.5	−17.3	−7.9	−15.6
Sodium, mg/100 g	−12.8	−10.1	−10.3	−5.8	−9.1
Magnesium, mg/100 g	−3.5	−3.2	−4.2	−3.4	−4.4
Calcium, mg/100 g	0.2	−0.4	−0.8	--	−0.2
Zinc, mg/100 g	−0.2	−0.3	−0.3	−0.4	−0.5
Iron, mg/100 g	−0.2	−0.1	--	−0.3	−0.3
Copper, mg/100 g	--	--	0.1	--	--
Selenium, µg/100 g	2.5	0.5	2.6	−0.1	−0.2

^1^ Strip loin steaks, tenderloin steaks, top sirloin steaks, and ribeye steaks were pan-grilled and rib roast was oven-roasted. ^2^ Nutrient values for cooked separable lean portions were adjusted based on a 23.6, 24.8, 25.3, 23.9, and 25.4% cook loss by weight to be compared to raw nutrient values on a 100 g basis.

## Data Availability

Data available by request due to technical and time limitations.
